# Differential expression of the angiotensin receptors (AT1, AT2, and AT4) in the placental bed of HIV-infected preeclamptic women of African ancestry

**DOI:** 10.1038/s41440-023-01314-x

**Published:** 2023-06-12

**Authors:** Shoohana Singh, Jagidesa Moodley, Thajasvarie Naicker

**Affiliations:** 1 Optics and Imaging Centre, Doris Duke Medical Research Institute, College of Health Sciences, University of KwaZulu-Natal, Durban, South Africa; 2 Women’s Health and HIV Research Group, Department of Obstetrics and Gynaecology, Nelson R. Mandela School of Medicine, College of Health Sciences, University of KwaZulu-Natal, Durban, South Africa

**Keywords:** Renin-Angiotensin Aldosterone System (RAAS), Preeclampsia, Angiotensin II-type 1 receptor (AT1R), Angiotensin II-type 2 receptor (AT2R), Angiotensin II-type 4 receptor (AT4R)

## Abstract

The Renin-Angiotensin-Aldosterone System (RAAS) is implicated in the pathophysiology of preeclampsia (PE). There is a paucity of data on uteroplacental angiotensin receptors AT1-2 and 4. We evaluated the immunoexpression of AT1R, AT2R, and AT4R within the placental bed of PE vs. normotensive (N) pregnancies stratified by HIV status. Placental bed (PB) biopsies (*n* = 180) were obtained from N and PE women. Both groups were stratified by HIV status and gestational age into early-and late onset-PE. Immuno-labeling of AT1R, AT2R, and AT4R was quantified using morphometric image analysis. Immunostaining of PB endothelial cells (EC) and smooth muscle cells of spiral arteries (VSMC) displayed an upregulation of AT1R expression compared to the N group (*p* < 0.0001). Downregulation of AT2R and AT4R expression was observed in PE vs. N group (*p* = 0.0042 and *p* < 0.0001), respectively. AT2R immunoexpression declined between HIV+ve and HIV−ve groups, while AT1R and AT4R displayed an increase. An increase in AT1R expression was noted in the EOPE−ve/+ve and LOPE−ve/+ve compared to N−ve/N+ve. In contrast, AT2R and AT4R expression decreased in EOPE−ve/+ve and LOPE-ve/+ve compared to N−ve/N+ve. We demonstrate a significant downregulation of AT2R and AT4R with a concomitant elevated AT1R immunoexpression within PB of HIV-infected PE women. In addition, a decline in AT2R and AT4R with an increase in AT1R immunoexpression in PE, EOPE, and LOPE vs. normotensive pregnancies, irrespective of HIV status. Thus highlighting differential immunoexpression of uteroplacental RAAS receptors based on pregnancy type, HIV status, and gestational age.

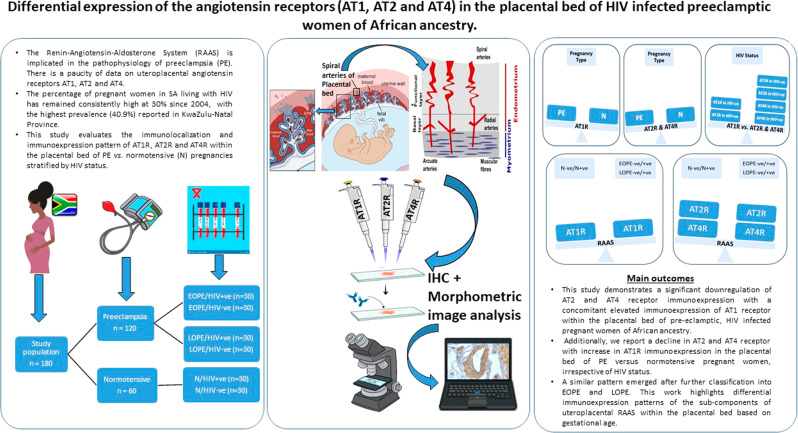

## Introduction

Human immunodeficiency virus (HIV) infection occurs in 13.9% of the overall population in South Africa (SA) [1]. In addition, the percentage of pregnant women in SA living with HIV has remained consistently high at 30% since 2004, with the highest prevalence (40.9%) reported in KwaZulu-Natal Province [2].

Preeclampsia (PE) is the most common direct cause of maternal mortality in SA and accounts for 88 deaths per 100,000 live births [3]. It is a pregnancy specific disorder characterized by raised blood pressure (systolic blood pressure ≥ 140 mm Hg or diastolic blood pressure ≥90 mm Hg at ≥20 weeks of gestation) at ≥20 weeks gestation [4]. In preeclampsia, water retention, proteinuria and progressive multi-organ injury are triggered by hypoxic injury predisposing the local and systemic production of anti-angiogenic and inflammatory factors [5, 6].

For placentation to be successful, trophoblast invasion facilitates the physiological remodelling of spiral arteries causing a substantial increase in arterial diameter [7]. This dilation accommodates an increase in blood flow to the developing fetus. In PE however, this transformation is limited to the decidua.

Pre-eclampsia may be classified by gestational age into early-onset PE (EOPE, <34 weeks gestation) which is associated with defective placentation, a decrease in placental volume, abnormal uterine and umbilical artery Doppler evaluation, multi-organ dysfunction, intra-uterine growth restriction, low birth weight and perinatal death [8]. In contrast, late-onset PE (LOPE, >34 weeks gestation) is classified as a maternal disorder. It is frequently linked to normal placentation, increased placental volume, normal uterine and umbilical artery Doppler evaluation, normal fetal growth and birth weight [8].

Hypertension is a resultant clinical indicator intricately controlled by the renin-angiotensin-aldosterone system (RAAS) [9]. In pregnancy, the RAAS preserves a fragile balance between the mother and fetus, who both contribute to the signalling cascade. The renal cortical juxtaglomerular cells release renin, a biomolecule of RAAS, in response to a decrease in effective blood flow, an increase in sympathetic tone, and renal artery stenosis. Renin’s proteolytic cleavage action splits angiotensinogen (AGT) into angiotensin I (Ang I). Angiotensin converting enzyme (ACE) catalyses the conversion of Ang I to form the powerful vasoconstrictor, Angiotensin II (Ang II). Ang II not only causes sodium and water retention but also aldosterone secretion which accelerates systemic vascular resistance, and antagonizes nitric oxide (NO), a potent vasodilator [10–12]. Modifications in the circulating RAAS during pregnancy is well-documented [13–15], but examining the uteroplacental RAAS is a challenge due to the invasive nature of obtaining biopsy samples at any time-point other than at caesarean delivery or early termination of the pregnancy. Renin and AGT levels are often elevated, resulting in higher levels of Ang II and aldosterone. This is a compensating strategy to mimic a non-pregnant woman’s vasomotor response. While pregnancy causes a drop in ACE [16], the plasma levels of Ang-(1-7) are higher in normotensive pregnancies compared to non-pregnant individuals however are lower in PE compared to normotensive pregnancies. This demonstrates that the development of hypertension in PE is compatible with the decreased plasma angiotension-1-7 [Ang-(1-7)] and Ang II [17, 18]. Angiotensin II type 1, type 2, and type 4 (AT1R, AT2R, and AT4R) expression is necessary for Ang II, to execute its physiological effects. Together, these receptors regulate renal, neurological, endothelial, cardiovascular, and cell proliferation and survival as well as inflammation. While AT2R and AT4R are linked to vasodilation, AT1R is associated with vasoconstriction [11, 19]. All three trimesters of pregnancy exhibit placental tissue expression of the AT1 receptor [20]. The severity of PE is correlated with this increased expression, suggesting that the enhanced expression may play a role in the pathophysiology of this pregnancy condition [21–23].

The AGT/renin/ACE/Ang II/AT1R axis is responsible for the vasoconstrictive, proliferative, and inflammatory effects whilst its de-regulation is linked to cardiovascular pathology, renal disease and hypertension [24]. Notably, the Ang II/aminopeptidase A (APA)/Ang III/AT2R/nitric oxide (NO)/cyclic guanosine monophosphate (cGMP) alliance serves as the RAAS vasodepressor and the cardio-renal protective axis, combating the negative effects of the RAAS pathway [24]. Initially, AT4R was described as a specific, high-affinity binding site for the hexapeptide angiotensin IV (Ang IV), but subsequently identified as a transmembrane enzyme, insulin-regulated membrane aminopeptidase (IRAP) in the Ang III/ aminopeptidase N (APN)/Ang IV/IRAP/AT4R pathway. This axis plays a crucial part in memory, blood flow control, and vasodilation [24].

Loss of endothelial cell (EC) integrity occurs in both PE and in HIV infection [25]. Changes in blood flow and elevation of interleukin-6 (IL-6), interleukin-8 (IL-8), tumor necrosis factor α (TNF-α), intercellular adhesion molecule-1 (ICAM-1), vascular cell adhesion molecule-1 (VCAM-1), monocyte chemoattractant protein-1 and von Willebrand factor (vWF) contribute to EC injury in HIV infected patients of African ancestry [26]. Strong angiogenic factors, such as the integrins V5, V1, and V3, fibronectin, and vitronectin, are bound by the HIV-1 transactivator of transcription (Tat) protein. In addition, it binds to Flk-1/KDR, Flt-1, and MAP kinase, which affects EC invasion, proliferation, and angiogenesis. Furthermore, the mechanism of HIV proteins—Tat, negative factor (Nef) and glycoprotein 120 (gp120) action leads to apoptotic resistant ECs/pro-survival state/angiogenesis/ elevated reactive oxygen species/ decreased eNOS expression and NO resulting in vasoconstriction and vascular remodelling [27]. Several complement components are increased with HIV infection and PE, resulting in a hyper inflammatory state [28]. The shared pathogenic features of HIV infection and PE may result from an overly active complement [29–32]. Untreated HIV infection was found to have a protective effect against preeclampsia prior to the widespread implementation of antiretroviral therapy in a setting with a high prevalence of HIV and preeclampsia. The protective effect against preeclampsia was not seen for HIV infection treated with antiretroviral therapy [33, 34].

The effect of ART such as protease inhibitors and non-nucleoside reverse transcriptase inhibitors may affect the RAAS. Evidence, however, indicates that adherence to cART increases the risk of hypertension development [35]. Studies in Cameroon report that 36.4% of HIV-positive non-pregnant patients receiving treatment, developed hypertension compared to 13.3% of treatment naïve HIV-positive patients [36]. It is now construed that elevated plasma renin activity in HIV-infected individuals result from enhanced RAAS upstream activation [37]. The structural homology between renin and HIV-1 protease further promotes plasma renin activity [38]. In addition, PE development in women using cART has been reported [39].

Our study aims to investigate the expression of AT1R, AT2R and AT4R in the placental bed tissue of pre-eclamptic HIV+ve South African women of African ancestry, utilizing immunohistochemistry and morphometric image analysis.

## Materials and methods

This prospective study was performed during the period November 2021–November 2022.

### Ethical considerations

This cross-sectional study received institutional ethics approval BE3257/2021 for use of archived wax embedded placental bed samples obtained in a primary study (BE: 040/12) and subsequently collated into a class approval (BCA338/17).

### Study population

The Cohen’s formula was used to determine the sample size. This study examined 180 wax embedded placental bed samples stratified by pregnancy type (normotensive, *n* = 60 vs. preeclamptic, *n* = 120) to detect a small effect size of 0.44. The preeclamptic group was sub-divided into PE onset (early-onset PE vs. late-onset PE; *n* = 60 per group) to detect a moderate effect size of 0.51. Lastly, all groups were further stratified by HIV status (HIV− and HIV+; *n* = 30 per group) to detect a moderate effect size of 0.7.

The study population consisted of primigravid and multigravid pre-eclamptic and normotensive pregnant women of African ancestry attending the maternity unit of a regional hospital in Durban, South Africa. The CD4 counts for all HIV+ women was determined as per SA’s Universal Test and Treat (UTT) policy and first-line antiretroviral treatment (ART) was initiated irrespective of CD4 cell count [40]. All women received a standardized HIV-drug regimen to treat HIV-infected pregnant women (regardless of CD4 count) during pregnancy and breastfeeding, with continuation of ART after breastfeeding for women with CD4 counts <350. The ARV treatment that was administered to women were either a single drug such as Zidovudine also known as Azidothymidine (AZT) or a combination of multiple drugs [Tenofovir disoprovil fumarate (TDF, Viread), Emtricitabine (FTC, Emtriva) and Efavirenz (EFV)]. The alternative drug combination administered to some of the patients was [Abacavar (ABC, Ziagen), Lamivudine (3TC, Epivir) and Efavirenz (EFV)] and PMTCT (Nevirapine) as per South African National HIV guidelines. HIV-exposed infants received Nevirapine prophylaxis for 4–6 weeks. Unfortunately, data on the duration of HIV infection remains unknown. The samples utilized were archived wax embedded samples and this information was not collated. None of the women from which placental bed specimens were obtained presented with HIV nephropathy or cardiac myopathy. HIV testing was done as routine to establish status as preeclampsia was the preferential criteria for selection.

The placental bed samples were collected at caesarean section using a curved scissor/scalpel biopsy method [41]. Biopsy of a true placental bed included extravillous trophoblasts and the presence of at least one spiral artery.

### Inclusion criteria

Pregnant women ≥18 years of age. Pre-eclampsia was defined as sustained systolic blood pressure ≥140/90 mmHg and/or a sustained diastolic blood pressure ≥90 mmHg taken on two occasions at least 6 h apart and proteinuria (+2 on the urine dipstick analysis) or >300 mg protein concentration in a 24 h urine specimen [4]. The study population was stratified into EOPE and LOPE sub-groups for pre-eclampsia [8].

### Exclusion criteria

Women with unknown HIV status, history of smoking and substance abuse, chronic hypertension, diabetes mellitus, gestational diabetes, epilepsy, chronic renal disease, connective tissue disease, heart failure, treatment with aspirin, warfarin, non-steroidal anti-inflammatory drugs, antibiotics lipid lowering or anti-hypertensive drugs, abruption placentae or intra-uterine death, chorioamnionitis, systemic lupus erythematosus, sickle cell disease, anti-phospholipid antibody syndrome, thyroid disease, active asthma requiring medication during pregnancy were excluded.

### Methods

Wax embedded placental bed samples were used for this study. These samples were initially fixed in neutral buffered formalin (10%) and processed as per standard laboratory procedure [42]. Sections of placental bed tissue were cut (3 µm) using a Leica rotary microtome (Leica LM 2135, Leica Biosystems, Germany) and floated onto coated slides (HistoBond X-tra Adhesive, Paul Marienfeld GmbH &Co.KG, Germany). Samples were de-paraffinized and rehydrated.

### Immunostaining

Heat induced antigen retrieval (HIAR) was performed for 10 min with citrate buffer (pH6.0) and cooled for a further 15–20 min with agitation to room temperature. Endogenous peroxidase was blocked with 2% hydrogen peroxide for 30 min followed by washing with Dako EnV Flex wash buffer (# K800721-2, Dako, CA) and a further background block was performed with SNIPER (# BC-BS966H, Biocare Medical, CA) for 30 min. Sections were buffer washed and followed by a final protein block for 30 min. A buffered wash followed by incubation in the polyclonal primary antibodies; AT1R (1 µg/ml, # PA5-20812, Invitrogen, MA), AT2R (2 µg/ml, # PA5-20813, Invitrogen, MA) and AT4R (1:200, # PA5-23777 [LNPEP], Invitrogen, MA), overnight in a humidity chamber at 4 °C. Antibody diluent (Dako REAL antibody diluent – # S202230-2, Dako, CA) was substituted for primary antibodies as method controls. A Rabbit specific HRP/DAB Micro-polymer IHC Detection Kit (# ab236469, Abcam, Bristol, United Kingdom) was used according to manufacturer’s instruction, with Goat anti-rabbit HRP conjugate serving as the secondary antibody. Visualization of the antibody’s adherent sites was achieved with chromogen 3.3′-Diaminobenzidine (DAB). Nuclei was counterstained with Shandon Haematoxylin (# 6765015, Thermo Scientific, MI) followed by dehydration, clearing and mounting in dibutylphthalate polystyrene Xylene (DPX).

### Morphometric analysis

The ApoTome 2 microscope (Carl Zeiss, Germany) was used to view the immunostained placental bed sections and a random selection of four fields of view per slide was analyzed at an initial objective magnification of 20X. ZEN Blue 2.5 Pro Software (Carl Zeiss, Germany) was used to optimize and acquire the images. The image analyzing software FIJI/ImageJ (Madison, WI) [43, 44] was used as a quantitative measuring tool. AT1R, AT2R and AT4R immuno-expression was evaluated as a percentage of immunostaining (region of interest) per total area of tissue.

### Statistical analysis

Prior to analysis, the results were examined for normality using the D’Agostino and Pearson, Shapiro–Wilk, and Kolmogorov–Smirnov tests. In order to compare the effects of pregnancy type (normotensive vs. preeclamptic) and HIV status (HIV−ve vs. HIV+ve) across all groups, a one-way ANOVA analysis with Kruskal–Wallis test was used. The data between subcategories was further evaluated using the Dunn’s multiple comparisons test. Statistical significance was defined as a probability threshold of *p* = 0.05. GraphPad Prism 8.4.3 (San Diego, CA) was used for all statistical analyses.

## Results

### Clinical characteristics

Patient demographics and clinical traits are shown in Table 1 as median and interquartile range (IQR) due to their non-parametric distribution. Maternal age (*p* < 0.0001), maternal weight (*p* = 0.0221), systolic pressure (*p* < 0.0001), diastolic pressure (*p* < 0.0001), gestational weight (*p* < 0.0001) and gestational age (*p* < 0.0001) were significantly different across study group.

**Table 1:** Patient demographics in normotensive HIV negative (N−ve); normotensive HIV positive (N+ve); Early-onset PE/HIV negative (EOPE−ve); Early-onset PE/HIV positive (EOPE+ve); Late-onset PE/HIV negative (LOPE−ve) and Late-onset PE/HIV positive (LOPE+ve) pregnant women

Total *n* = 180	N−ve	N+ve	EOPE−ve	EOPE +ve	LOPE −ve	LOPE+ve	*p* value and Dunn’s Multiple comparison	
	*n* = 30	*n* = 30	*n* = 30	*n* = 30	*n* = 30	*n* = 30		
Maternal Age (years)	25 (22–30)	28 (25–31)	23 (20–27)	32 (25–37)	23 (19–26)	28 (25–34)	**<0.0001** N+ve vs. LOPE−ve EOPE−ve vs. EOPE+ve EOPE−ve vs. LOPE+ve EOPE+ve vs. LOPE-ve LOPE−ve vs. LOPE+ve	**** * ** * *** **
Weight (kg)	73.70 (63.25–97.77)	67.30 (55.25–85.38)	67.20 (60.25–91.00)	81.00 (68.48–87.90)	66.00 (55.75–81.00)	85.35 (69.90–95.25)	**0.0221** LOPE−ve vs. LOPE+ve	* *
Systolic Pressure (mmHg)	111.0 (106.0–116.8)	111.0 (105.0–117.0)	151.0 (143.0–160.0)	148.0 (136.0–161.0)	154.0 (146.5–173.0)	145.0 (141.8–153.3)	**<0.0001** N−ve vs. EOPE−ve N−ve vs. EOPE+ve N−ve vs. LOPE−ve N−ve vs. LOPE+ve N+ve vs. EOPE−ve N+ve vs. EOPE+ve N+ve vs. LOPE−ve N+ve vs. LOPE+ve	**** **** **** **** **** **** **** **** ****
Diastolic Pressure (mmHg)	71.00 (64.50–79.00)	70.00 (64.00–76.00)	95.50 (91.00–104.3)	92.00 (90.00–98.00)	101.0 (94.00–108.0)	96.00 (91.00–98.25)	**<0.0001** N−ve vs. EOPE−ve N−ve vs. EOPE+ve N−ve vs. LOPE-ve N−ve vs. LOPE+ve N+ve vs. EOPE−ve N+ve vs. EOPE+ve N+ve vs. LOPE−ve N+ve vs. LOPE+ve	**** **** **** **** **** **** **** **** ****
Gestational weight (kg)	3.150 (2.975–3.585)	3.100 (2.600–3.550)	2.100 (1.625–2.538)	1.900 (1.300–2.500)	3.000 (2.723–3.213)	2.900 (2.300–3.120)	**<0.0001** N−ve vs. EOPE−ve N−ve vs. EOPE+ve N+ve vs. EOPE−ve N+ve vs. EOPE+ve EOPE−ve vs. LOPE−ve EOPE−ve vs. LOPE+ve EOPE+ve vs. LOPE−ve	**** **** **** **** *** **** * **
Gestational Age (weeks)	38.00 (37.00–39.00)	37.00 (36.00–39.00)	33.00 (30.00–35.00)	30.00 (25.75–34.50)	35.50 (34.00–37.00)	35.00 (33.00–36.00)	**<0.0001** N−ve vs. EOPE−ve N−ve vs. EOPE+ve N−ve vs. LOPE−ve N−ve vs. LOPE+ve N+ve vs. EOPE−ve N+ve vs. EOPE+ve N+ve vs. LOPE−ve N+ve vs. LOPE+ve	**** **** **** **** **** **** **** * **

Key: Results are represented as the median (IQR), *p < 0.05, **p < 0.01, ***p < 0.001 and ****p < 0.0001

Across the population group (*n* = 180), the Kruskal–Wallis (K-W) test detected significance for maternal age (*p* < 0.0001), weight (*p* = 0.0221), systolic and diastolic pressure (*p* < 0.0001), and gestational weight and age (*p* < 0.0001). The Dunn’s multiple comparison test further calculated significance between the PE groups stratified by HIV, indicated in Table 1

Bold values denote significant *p* values

### AT1R, AT2R and AT4R placental bed immunolocalization

**Fig. 1:**
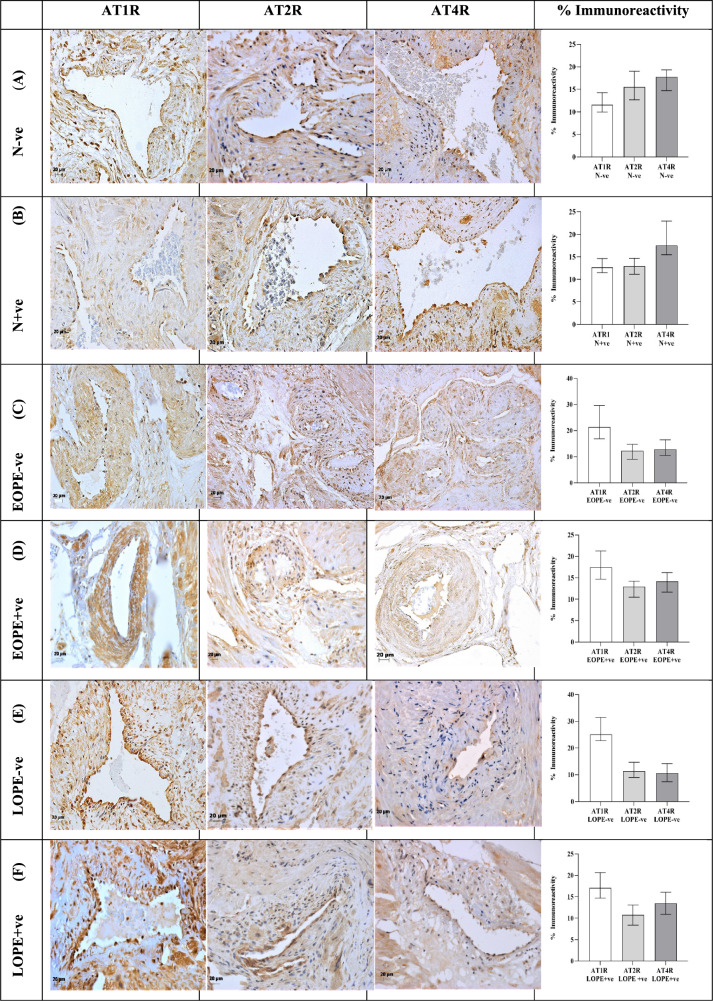
Micrographs of immunostained spiral arteries in placental bed tissue for AT1R, AT2R and AT4R of normotensive HIV negative (N-ve); normotensive HIV positive (N+ve)-[**A**,**B**]; Early-onset PE/HIV negative (EOPE−ve); Early-onset PE/HIV positive (EOPE+ve)-[**C**,**D**]; Late-onset PE/HIV negative (LOPE–ve) and Late-onset PE/HIV positive (LOPE+ve)- [**E**,**F**] pregnant women. Placental bed tissue sections are immune-labelled with (from left to right) AT1R, AT2R and AT4R, and counterstained with Haematoxylin (H). Representative images of (**A** and **B**) of spiral arteries from a normotensive (N) group with complete physiological transformation, characterized by the presence of AT1R, AT2R and AT4R-positive vascular endothelial cells (EC) and trophoblasts. The intramural fibrinoid (pale blue on H-staining) in the vessel wall, and complete absence of intramural smooth muscle cells. **C** and **D** Spiral arteries from the early-onset preeclampsia (EOPE) groups with unsuccessful physiological transformation (EC, tunica media of smooth muscle and trophoblasts [AT1R, AT2R and AT4R positive]). **E** and **F** Spiral arteries from the late-onset preeclampsia (LOPE) groups with partial physiological transformation (EC, tunica media of smooth muscle and trophoblasts [AT1R, AT2R and AT4R positive] and presents intramural fibrinoid

**Fig. 2:**
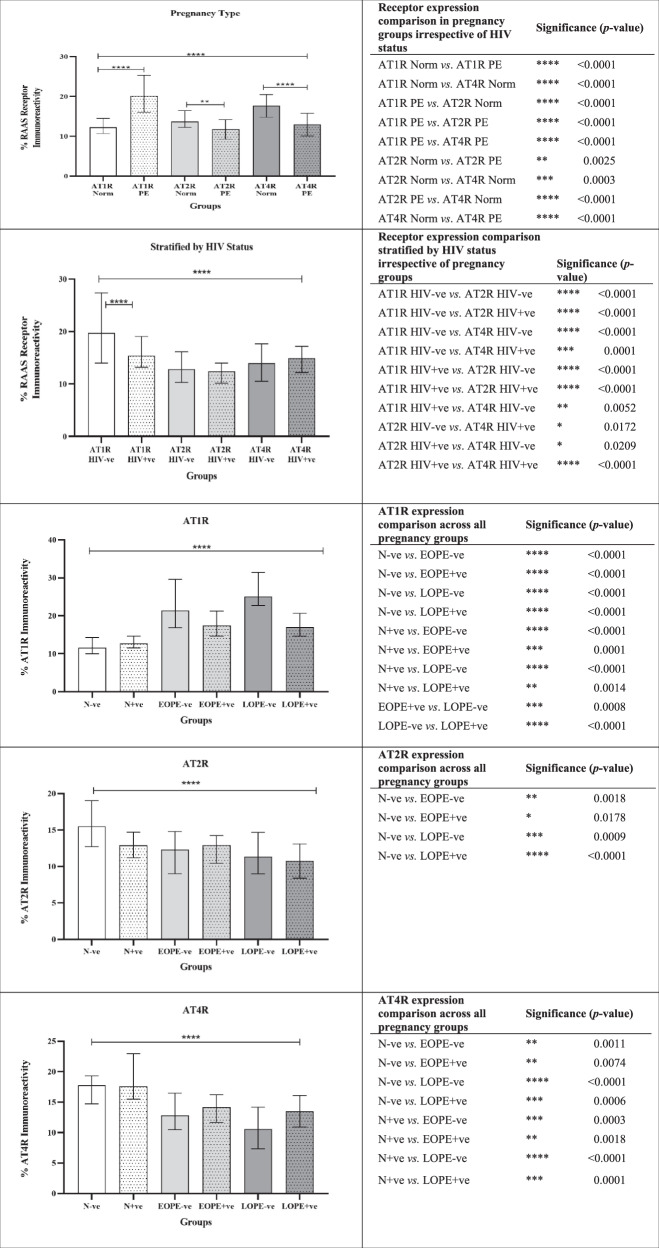
Graphs representing morphometric image analysis of immunostained placental bed tissue in normotensive HIV negative (N−ve); normotensive HIV positive (N+ve); Early-onset PE/HIV negative (EOPE−ve); Early-onset PE/HIV positive (EOPE+ve); Late-onset PE/HIV negative (LOPE−ve) and Late-onset PE/HIV positive (LOPE+ve) pregnant women

AT1R, AT2R and AT4R immunostaining within the spiral arteries of the placental bed was evident in all subgroups *viz*., N+ve, N−ve, EOPE−ve, EOPE+ve, LOPE−ve and LOPE+ve. Immunolocalization of all three receptors was observed in EC and trophoblasts and fibroblast like cells embedded within the fibrinoid of spiral arteries as well as the mild immunostaining of VSMC of the PE group as indicated in Fig. 1. Corresponding morphometric image analysis data is represented in Fig. 2.

### Pregnancy type

The field area percentage of AT1R expression showed a significant increase in the PE (20.10%, 16.07–25.30%) compared to the normotensive groups (12.28%, 10.69–14.49% [*p* < 0.0001]). Significant downregulation of AT2R was observed in PE (11.77%, 9.27–14.18%) compared to the normotensive groups [13.71%, 12.27–16.46% (*p* = 0.0042)]. A significant decline in AT4R expression was also detected in the PE (13.00%, 10.09–15.76%) vs. the normotensive groups [17.70%, 14.79–20.48% (*p* < 0.0001)]. The Dunn’s multiple comparison test showed significant differences between AT1R, AT2R and AT4R expression across all groups (K-W = 281.5, *p* < 0.0001), indicated in Fig. 2.

### HIV status

The field area percentage of AT1R expression showed a non-significant downregulation in the HIV+ve (15.40%, 13.19–19.07%) compared to the HIV-ve groups (19.76%, 13.99–27.36%). A minimal non-significant decline in expression of AT2R was observed in HIV+ve compared to the HIV-ve groups (12.45% vs. 12.81%). In contrast, AT4R expression was mildly increased in the HIV+ve compared to HIV-ve groups (14.95% vs. 14.00%) albeit not significantly. The Dunn’s multiple comparison test showed significant differences between AT1R HIV−ve and AT1R HIV+ve compared to AT2R HIV-ve/HIV+ve and AT4R HIV−ve/HIV+ve expression (K-W = 119.4, *p* < 0.0001), indicated in Fig. 2.

### AT1R across pregnancy groups stratified by HIV

Placental bed of the N+ve group displayed a non-significant decrease in AT1R expression in comparison to the N−ve group (12.68% vs. 11.57%). Similarly, the EOPE+ve group showed a moderate downwards trend compared to the EOPE−ve group (17.43% vs. 21.35%). However, the LOPE+ve (17.05%, 14.66–20.64%) demonstrated a significant downregulation of AT1R expression compared to the LOPE-ve groups [25.04%, 22.73–31.44% (*p* < 0.0001)]. The Dunn’s multiple comparison test showed significant differences in expression of AT1R between N−ve vs. EOPE−ve/EOPE+ve/LOPE−ve/LOPE+ve and N+ve vs. EOPE−ve/EOPE+ve/LOPE−ve/LOPE+ve as well as LOPE-ve vs. EOPE+ve/LOPE−ve groups (K-W = 139.9, *p* < 0.0001), with corresponding *p* values noted in Fig. 2.

### AT2R across pregnancy groups stratified by HIV

The percentage immunoreactivity of AT2R in the N+ve and LOPE+ve groups demonstrated a decline compared to the N−ve and LOPE−ve, respectively, albeit non-significant (12.90% vs. 15.50% and 10.76% vs. 11.33%). In contrast, the EOPE+ve group showed a mild, non-significant up-regulation in AT2R expression than the EOPE−ve group (12.93% vs. 12.31%). The Dunn’s multiple comparison test showed significant differences in expression of AT2R between N−ve vs. EOPE−ve/EOPE+ve/LOPE−ve/LOPE+ve (K-W = 29.88, *p* < 0.0001), with corresponding *p* values indicated in Fig. 2.

### AT4R across pregnancy groups stratified by HIV

A generalized increased trend of AT4R expression was noted in the N+ve/EOPE+ve/LOPE+ve compared to the N−ve/EOPE−ve/LOPE−ve (17.57%/14.15%/13.49% vs. 17.76%/12.83%/10.59%), respectively. A significant decrease in expression of AT4R was observed in the EOPE –ve/+ve and LOPE−ve/+ve groups vs. the N+ve and the N−ve groups (K-W = 65.82, *p* < 0.0001), with corresponding *p* values listed in Fig. 2.

## Discussion

The main finding of this study show the variance in immunoexpression of the RAAS receptors AT1, AT2, and AT4 in the placental bed spiral arteries of South African women of African ancestry, stratified by pregnancy type (Normotensive and preeclamptic), gestational age (EOPE and LOPE), and co-morbid HIV infection (HIV−ve and HIV+ve).

### AT1R

This study demonstrated the immunolocalization of AT1R within ECs and vascular smooth muscle cells, embedded trophoblast cells in the fibrinoid of spiral arteries as well as interstitial trophoblasts across all pregnancy groups. Our results show significantly elevated expression of AT1R in PE compared to N group regardless of HIV status. These findings are corroborated by a similar significant increase of this potent vasopressor receptor in PE compared to the normotensive group, irrespective of HIV status [45]. This amplified AT1R immunoexpression would naturally compete for the low levels of circulating Ang II in PE [46]. Angiotensin II is a powerful mediator for oxidative stress and oxidant signaling. The binding of Ang II to AT1R stimulates membrane nicotinamide adenine dinucleotide phosphate (NADPH) oxidase to produce reactive oxygen species (ROS) like superoxide, hydrogen peroxide, thereby promoting inflammation, altering vasoreactivity, EC growth, migration, platelet activation, and fibrosis [47]. Activation of AT1R elicits a powerful effect on vascular smooth muscle cells facilitating its constrictor, migratory, growth-promoting, proliferative, and apoptotic effects. Vascular remodeling, inflammation, fibrosis, and atherosclerosis are pathophysiological effects of excessive AT1R activation [48]. The root cause of endothelial dysfunction in hypertension, atherosclerosis, and cardiovascular disorders is the inactivation of nitric oxide by AT1R-induced ROS [49, 50]. Hypertension, atherosclerosis, thrombosis, chronic renal disease, and insulin resistance, eventually lead to the development of cardiovascular disease [5]. In our study, the clinical outcome of the AT1R upregulation in the PE group was corroborated by the increased blood pressure in PE.

In addition, oxidative stress is a trigger for leukocyte activity, allowing for lymphocytes and monocyte/macrophages to express all RAAS components and have the capacity to produce local Ang II in the tissues they invade, such as blood vessels of placental bed [51]. According to several recent studies, an agonistic autoantibody to the AT1R (AT1-AA) exists in PE suggesting that AT1-AA, through activation of NADPH oxidase, could contribute to ROS production and the inflammatory responses associated with PE [52–54]. Furthermore, activation of AT1R by AT1-AA on human trophoblasts may contribute to increased plasminogen activator inhibitor (PAI-1) production thereby inhibiting trophoblast invasion in PE [54]. AT1R signaling boosts the production of Transforming growth factor-β1, laminin, and fibronectin and promoting enhanced fibroblast attachment to collagen types I and III as well as increased focal adhesion kinase activity, mediating profibrotic effects of tissue [55]. AT1R receptor activation stimulates trans-activating epidermal growth factor receptor-mitogen-activated protein kinase (EGFR-MAPK) dependent signaling pathways with resultant dysregulated buildup of proteoglycans emanating from extracellular matrix protein synthesis in atherosclerotic lesions [56]. In combination, these factors contribute to the unsuccessful physiological transformation of spiral arteries. This feature was demonstrated in our study with the normotensive group displaying total physiological transformation, however the LOPE and EOPE groups exhibited partial to absence of myometrial physiological transformation, respectively.

In contrast, the HIV+ve group showed a significant downregulation of AT1R expression compared to the HIV-ve group, regardless of pregnancy type. Based on pregnancy type and HIV status, the N+ve exhibited a moderate decline in AT1R expression compared to N−ve group. However, EOPE-ve/+ve and LOPE−ve/+ve showed overall elevated levels of AT1R expression compared to the N−ve/+ve groups. Notably, the EOPE+ve and LOPE+ve groups showed a decline in expression compared to the EOPE−ve and LOPE−ve, but not lower than the N−ve/+ve groups. These results highlight the effect of the duality of HIV status and gestational age (EOPE or LOPE) on AT1R expression. An impaired maternal immune response or inadequate maternal tolerance to the fetus are two common theories to explain deficient placentation in PE [31].

As immunological hyperactivity may be neutralized, conditions of acquired immunodeficiency like HIV infection could prevent the development of PE. There are conflicting reports of prevalence and risk factors in relation to PE co-morbid with HIV [35]. The initiation of cART leads to immune restoration [57]. HIV is able to block endogenous anti-oxidant enzymatic systems [58], with consequential elevated systemic oxidative stress [59]. Furthermore, placental syncytiotrophoblasts express the retroviral gene syncytin and PE pathogenesis may be related to the de-regulation of this viral fusion protein of endosymbiotic endogenous retroviruses [60]. Moreover, the HIV-1 accessory proteins *tat*, gp120, and *nef* are responsible for EC injury [61]. In HIV infection, changes in blood flow with concomitant increase in the circulation of biomarkers like IL-6, D-dimer, fibrinogen, C-reactive protein, TNF-α, soluble intercellular adhesion molecule (sICAM), soluble vascular cell adhesion molecule (sVCAM), and endothelial microvesicles exacerbate EC injury and loss of integrity [62].

The HIV-1 tat protein, a potent angiogenic factor, promoting EC growth, angiogenesis and invasion [63]. Endothelial cell dysfunction is triggered when tat proteins are released from infected cells, including receiving cART [64]. cART use is linked to a higher prevalence and fatality rates among PE cases [25]. Adherence to cART raises the risk of preterm birth in the synergy of pregnancy and HIV infection [65, 66].

### AT2R

In adults, AT2R are expressed in several tissues including heart, kidney, adrenal gland, brain, skin, ovary, uterus and both endothelial and vascular smooth muscle cells [67]. AT2R-mediated vasorelaxation in mesenteric, renal, coronary, cerebral and placental bed vasculature, is elicited via stimulation of NO/cyclic guanosine monophosphate (cGMP)/ big potassium (BK) signaling pathways [68, 69]. Thus it has efficaciously earning the title of “protective arm” in the RAAS by mediating tissue-protective actions such as anti-inflammation, anti-fibrosis, and anti-apoptosis [69]. Our study showed the competitive receptor for Ang II, AT2R was significantly downregulated in placental bed vasculature of PE compared to the N group, irrespective of HIV status. This decline was also noted between AT2R and AT1R for both PE and N groups, implicating the counteractive vasodilation and tissue-protective capabilities of the AT2R was insufficient to counteract the action of AT1R.

We report a non-significant decline of AT2R expression in the HIV+ve group compared to the HIV-ve group, regardless of pregnancy type. Examining the pregnancy groups based on HIV status, the EOPE−ve, EOPE+ve, LOPE−ve and LOPE+ve exhibited a significant downregulation in AT2R expression compared to N−ve group. Although a decline in expression of AT2R was evident in the EOPE−ve and EOPE+ve groups compared to N−ve, both EOPE−ve/+ve were mildly higher than LOPE−ve/+ve, albeit non-significant. Our finding is corroborated with reports suggesting that RAAS activation and chronic immunological activation play a role in the emergence of the metabolic syndrome and hypertension in HIV-positive individuals [70].

It is also plausible that women are susceptible to PE development based on single nucleotide polymorphisms of AT1R and AT2R. This outcome was observed in PE and normotensive Iranian women where the AC/AG combination significantly decreased, whereas the CC/AA combination significantly increased, suggesting that Iranian women’s vulnerability to PE may be influenced by genetic variations within the AT1R and AT2R genes [71]. However, this variation was not identified in women of African ancestry as genotype distributions of Renin (REN), AT1R, AT2R angiotensin converting enzyme (ACE) were similar in PE and normotensive groups. The only difference was noted for angiotensinogen (AGT) where the distribution of T allele and TT genotype in PE were significantly higher than the normotensive group and aldosterone synthase, especially in those without HIV infection may prevent them from developing PE [72, 73].

### AT4R

Similar to the AT1R and AT2R of the RAAS, AT4R is located in several tissue types, including brain, VSMCs, vaso vasorum, within small angiogenic vessels, and invariably in the heart, kidney and adrenal glands, [74, 75]. Ang II was formerly considered as the principal bioactive peptide for AT4R, but it has recently come to light that Ang IV, which emanate from a two-step aminopeptidase (A and N) enzyme cleavage of Ang II, participates in controlling cardiovascular function [76]. It appears that Ang IV works in multiple tissues with high binding affinity to its preferred receptor AT4. The insulin-regulated aminopeptidase (IRAP), also known as placental leucine aminopeptidase (P-LAP), has been identified as the active site for AT4R [77]. Both endothelial and smooth muscle cells express AT4R, indicating that these cells play physiological roles in controlling blood flow. AT4R via NO-mediated mechanism enables Ang IV thereby improving blood flow. The presence of this receptor in extravillous trophoblast raises the possibility that it plays a role in controlling EVT invasion during placentation.

In our study, a significant decline in AT4R expression was detected in the PE vs. N groups, irrespective of HIV status. Furthermore, AT4R expression was mildly increased in the HIV+ve compared to HIV-ve groups with no significance, regardless of pregnancy type. Examining the pregnancy groups based on stratification by HIV, revealed that the EOPE−ve, EOPE+ve, LOPE−ve and LOPE+ve exhibited a significant downregulation in AT4R expression compared to N−ve and N+ve groups. Placental bed research in combination with AT4R is limited, however similar outcomes in circulating plasma levels of soluble AT4R where women with severe features of PE had lower levels than normotensives was shown [78]. Gene polymorphism study of AT4R and aminopeptidase-N did not significantly associate with PE for in South African women of African ancestry [79].

## Strengths and limitations of the study

To our knowledge, this is the first prospective cohort study to evaluate the triad of AT1R, AT2R and AT4R immune-expression on myometrial spiral arteries of placental bed tissue of PE and normotensive pregnancies stratified by gestational age and HIV infection. Due to the invasive nature of placental bed biopsies, the sample size was small.

## Conclusion

The culmination of our study highlights a significantly attenuated expression of AT2R and AT4R compared to AT1R at the placental bed level in the synergy of HIV infection of pregnant women of African ancestry. However, preeclamptic women exhibited a significant decline in AT2R and AT4R expression as opposed to an increased AT1R immune reactivity at the placental bed level, regardless of HIV status. Further categorization into early and late on-set PE showed a similar trend. This study provides evidence that uteroplacental based RAAS dysregulation contributes to abnormal angiogenic vasodilation in PE development.
